# Investigating smoking and nicotine dependence among people with severe mental illness during the COVID-19 pandemic: analysis of linked data from a UK Closing the Gap cohort

**DOI:** 10.1192/bjo.2021.45

**Published:** 2021-04-23

**Authors:** Emily Peckham, Victoria Allgar, Suzanne Crosland, Paul Heron, Gordon Johnston, Elizabeth Newbronner, Elena Ratschen, Panagiotis Spanakis, Ruth Wadman, Lauren Walker, Simon Gilbody

**Affiliations:** Mental Health and Addiction Research Group, University of York, UK; Hull York Medical School, UK; Mental Health and Addiction Research Group, University of York, UK; Mental Health and Addiction Research Group, University of York, UK; Mental Health and Addiction Research Group, University of York, UK; Mental Health and Addiction Research Group, University of York, UK; Mental Health and Addiction Research Group, University of York, UK; Mental Health and Addiction Research Group, University of York, UK; Mental Health and Addiction Research Group, University of York, UK; Mental Health and Addiction Research Group, University of York, UK; Mental Health and Addiction Research Group, University of York, UK

**Keywords:** Severe mental illness, smoking cessation, smoking, COVID-19, schizophrenia

## Abstract

Smoking rates are higher for people who use mental health services, which contributes substantially to health inequalities. Smoking can lead to worse COVID-19 outcomes, yet it remains unclear whether smoking has changed for people who use mental health services. We examined smoking patterns in a large clinical cohort of people with severe mental illness, before and during the pandemic. We found high levels of nicotine dependence and heavier patterns of smoking. Although some people had reported quitting, it is likely that smoking inequalities have become further entrenched. Mental health services should seek to mitigate this modifiable risk and source of poor health.

People with severe mental illness (SMI) experience profound health inequalities, with increased rates of long-term physical health problems and behavioural risk factors, such as smoking.^1^ There are concerns that the COVID-19 pandemic will disproportionately affect the health of people with SMI, increasing already existing health inequalities.^2^ Around 40% of people with SMI smoke,^3^ compared with a current smoking prevalence in England of around 14%.^4^ High rates of smoking remain an important modifiable risk factor, and there is good evidence that effective interventions can encourage successful quitting.^5^

The COVID-19 pandemic has affected health risk behaviours, and there has been an increase in quit attempts in the wider UK population, although the impact on smoking rates remains unclear.^6^ For example, in England, it has been reported that 300 000 people quit smoking^7^ during the first months of the pandemic. A national campaign urging people to ‘QuitForCOVID’ was rolled out, but it is uncertain whether people with SMI benefitted from this health promotion campaign. UK population surveys (e.g.^6^) have not been able to study this, and it remains possible that disparities in smoking and quitting have been further entrenched among people with SMI during the pandemic. Studies describing health risk behaviours among people with SMI provide an opportunity to explore changes during the COVID-19 pandemic. Here, we describe changes in smoking behaviour and quitting among people with SMI.

## Method

The Closing the Gap (CtG) study is a large (*n* = 10 176) clinical cohort, recruited between April 2016 and March 2020. Participants have documented diagnoses of schizophrenia or delusional/psychotic illness (ICD-10^8^ codes F20.X and F22.X or DSM IV or Vequivalent) or bipolar disorder (ICD-10 code F31.X or DSM equivalent). The composition of the CtG cohort has previously been described,^9^ and the data at inception included descriptions of self-reported smoking behaviour and e-cigarette use.

We aimed to explore the effects of the COVID-19 pandemic in a subsection of this clinical cohort, and we identified participants for the Optimising Well-being in Self-Isolation (OWLS) study (https://sites.google.com/york.ac.uk/owls-study/home). We aimed to recruit 300 people to the OWLS study, as this number was feasible in the timescale. To ensure that the OWLS study subcohort was representative, we created a sampling framework based on gender, age, ethnicity and whether they were recruited via primary or secondary care. OWLS study participants were recruited from 17 mental health trusts (in six clinical research networks areas across urban and rural settings in England). People who met the eligibility criteria were contacted by telephone or letter, and invited to take part in the study.

We explored smoking behaviour and e-cigarette use during the COVID-19 pandemic. For those who reported smoking or using e-cigarettes, we asked whether this was more, less or about the same since the pandemic restrictions began. Those who reported smoking also completed the Heaviness of Smoking Index (HSI), derived from the Fagerstrom Test for Nicotine Dependence^10^ (i.e. how many cigarettes per day they smoked and how long after waking they had their first cigarette), with categories of low, medium and high.

Changes in smoking status since completing the original CtG survey were established by participant linkage between self-reported smoking status before and after the pandemic restrictions began. We also compared smoking statistics within our SMI population with recent figures drawn from the UK-wide survey of smoking in the general population.^4^

### Ethics

We assert that all procedures contributing to this work comply with the ethical standards of the relevant national and institutional committees on human experimentation and with the Helsinki Declaration of 1975, as revised in 2008. All procedures involving human patients were approved by the North West – Liverpool Central Research Ethics Committee (reference 20/NW/0276). Written or verbal informed consent was obtained from all patients.

## Results

Between July and December 2020, 367 people with SMI were recruited to the OWLS study. The mean age was 50.5 (range 20–86) years, 51% were men and 77.4% were White British.

Smoking-related behaviour is given in Table 1. A total of 27.0% (95% CI 22.6–31.7%) of study participants reported that they smoked, compared with 14.1% of the general population,^4^ and 12.8% (95% CI 9.7–16.5%) of study participants reported that they used an e-cigarette. Participants who smoked reported smoking a mean of 17.5 cigarettes per day (minimum of 2 and maximum of 60), compared with 9.1 of the general population.^4^ With reference to the HSI, 54.5% of study participants reported smoking within 5 mins of waking, compared with 6.9% of the general population. In terms of the HSI, 50.5% of people were moderately addicted and 23.2% were highly addicted.

**Table 1:** Smoking behaviour, nicotine dependence and e-cigarette use in people with severe mental illness

Characteristic	OWLS study (*N* = 376), *n*, %
Percentage of people who smoke	99, 27.0% (95% CI 22.6–31.7%)
Mean number of cigarettes per day	17.5 (Interquartile range 10–20)
Percentage of people who smoked within 5 mins of waking^a^	54, 54.5% (95% CI 44.7–64.1%)
Heaviness of smoking^a^^,^^b^	
Low	21, 21.2% (95% CI 14.1–30.0%)
Medium	50, 50.5% (95% CI 40.8–60.2%)
High	23, 23.2% (95% CI 15.8–32.2%)
Percentage of people who use an e-cigarette	47, 12.8% (95% CI 9.7–16.5%)

OWLS, Optimising Well-being in Self-Isolation study.

a. Per cents are out of those who smoked.

b. Per cents do not add up to 100% because of non-responders.

Of the people who reported smoking in the OWLS survey, 54.5% (95% CI 44.7–64.1%) said that they had been smoking more heavily since the pandemic restrictions began, and 12.1% (95% CI 6.8–19.6%) said that they had been smoking less.

A total of 355 participants provided smoking data both in the OWLS study and in a pre-COVID-19 data collection. Longitudinal linkage with pre-COVID-19 data showed that, by self-report, 9.0% (95% CI 6.4–12.3%) of people with SMI had stopped smoking. However, 5.9% (95% CI 3.8–8.7%) of people had started smoking. This change in smoking status could have taken place at any time between April 2016 to March 2020 (initial data collection) and July to December 2020 (follow-up time period).

## Discussion

Smoking remains one of the most important modifiable risk factors for reduced life expectancy among people with SMI.^1^ To our knowledge, this is the first study to examine smoking status and smoking behaviour change for people with SMI during COVID-19, and other smoking population surveys have not been able to study this. Our study complements recent population surveys of mental health,^11^ which have either not been able to capture the experiences of people with SMI, or have focused on mental health and not health risk behaviours. Compared with when cohort participants were first surveyed pre-COVID-19, the smoking rate has dropped, which is encouraging and suggests that there have been successful quit attempts; however, we cannot be certain whether this change in smoking behaviour is a result of COVID-19 and the pandemic restrictions. We also noted that smoking intensity increased for people who currently smoke, with more than half reporting smoking more. This suggests that although the pandemic may have prompted some people to change their smoking behaviour, for those who continued to smoke, aspects of the pandemic restrictions may have led to them smoking more.

As anticipated, when compared with the general population,^4^ people were smoking on average about twice as many cigarettes per day as people without SMI, and people with SMI were nearly ten times more likely to report smoking within 5 mins of waking. This indicates that people with SMI who smoke are likely to have a higher level of nicotine dependence than those without an SMI, although we cannot link this to COVID-19. A limitation of the study is that data on nicotine dependence were not available pre-COVID-19. However, we propose to study temporal trends in smoking and nicotine dependence beyond the COVID-19 pandemic.

We conclude that important disparities between the wider population and people with SMI remain, and that smoking-related inequalities have potentially increased since the beginning of the COVID-19 pandemic. It is therefore important that effective quitting services are provided for, and responsive to, the needs of people who use mental health services.

## Data Availability

The data that support the findings of this study are available on request from the corresponding author, E.P.
